# The Impact of Hip Fracture on Geriatric Care and Mortality Among Older Swedes: Mapping Care Trajectories and Their Determinants

**DOI:** 10.1093/aje/kwac149

**Published:** 2022-08-15

**Authors:** Anna C Meyer, Marcus Ebeling, Sven Drefahl, Margareta Hedström, Stina Ek, Glenn Sandström, Karin Modig

**Keywords:** aging, elder care, hip fracture, home care, nursing homes, osteoporosis, registers, Sweden

## Abstract

In this study, we examined the impact of hip fractures on trajectories of home care, nursing home residence, and mortality among individuals aged 65 years or more and explored the impacts of living arrangements, cohabitation, frailty, and socioeconomic position on these trajectories. Based on a linkage of nationwide Swedish population registers, our study included 20,573 individuals with first hip fracture in 2014–2015. Care trajectories during the 2 years following the fracture were visualized and compared with those of 2 hip-fracture–free control groups drawn from the general population: age- and sex-matched controls and health-matched controls identified through propensity score matching. Multistate modeling was employed to identify sociodemographic and health-related factors associated with care trajectories among hip fracture patients. We found that hip fracture patients already had worse health than the general population before their fracture. However, when controlling for prefracture health, hip fractures still had a considerable impact on use of elder-care services and mortality. Comparisons with the health-matched controls suggest that hip fractures have an immediate, yet short-term, impact on care trajectories. Long-term care needs are largely attributable to poorer health profiles independent of the fracture itself. This emphasizes the importance of adequate comparison groups when examining the consequences of diseases which are often accompanied by other underlying health problems.

## Abbreviations

LISA: Longitudinal Integrated Database for Health Insurance and Labour Market Studies
NPR: National Patient Register
SSR: Social Service Register

Hip fractures are one of the most common causes of care dependency in old age, and numerous studies have demonstrated the severe impact of hip fractures on the physical and mental functioning of older individuals (1–4). In a comprehensive literature review, Dyer et al. (1) concluded that 1 in 2 hip fracture patients fail to regain their prefracture levels of mobility and independence. In addition to long-standing disability, hip fractures have been associated with decreased quality of life, as well as excess mortality persisting for several years after the fracture (1–9).

Most studies examining health-related consequences of hip fractures focus on mortality, functional impairment, or the risk of complications such as infections (9–13). Less is known about the impact of hip fractures on older individuals’ living arrangements and their need for long-term care, although these are important outcomes for both patients and welfare systems. Previous research has found that 10%–20% of community-dwelling individuals move to permanent care homes (equivalent to US nursing homes) after experiencing a hip fracture (1, 14, 15), but, to our knowledge, few studies present populationwide data on living arrangements before and after hip fracture or examine factors associated with patients’ living arrangements—especially including patients already residing in nursing homes. A substantial proportion of individuals who have sustained a hip fracture live in nursing homes, which has been associated with poorer recovery compared with living in the community (1, 15, 16). Moreover, most previous studies built upon clinical cohorts of hip fracture patients and their progress over time, without comparison with nonfracture control groups. Such comparisons are necessary, however, to isolate the effect of a hip fracture from the gradual declines in health that occur even in the absence of a hip fracture (1). At the same time, the prefracture health of individuals who sustain a hip fracture may be poorer than health in the general older population. Thus, a direct comparison of use of elder-care services among hip fracture patients and the general population may overestimate the impact of the hip fracture itself. Therefore, researchers should take into account patients’ health and care status prior to their fracture when examining the impact of hip fractures on later health outcomes.

Aside from prefracture living arrangements and underlying health profiles, previous research has identified several predictors for the prognosis of hip fracture patients, including treatment-related factors (2), socioeconomic position (16, 17), social support (16), and comorbidity (2, 9, 18–21), with several studies highlighting the role dementia plays in prognosis after hip fracture (9, 19–21). This demonstrates that hip fracture patients are a heterogeneous group with different recovery prospects, and that many of the factors linked to patients’ prognoses may also influence their elder-care use and living arrangements.

Building upon national health registers, our study aimed to examine the impact of hip fractures on long-term geriatric care, living arrangements, and mortality. We illustrate care trajectories in a nationwide cohort of hip fracture patients over the age of 65 years in comparison with matched population controls, as well as prefracture-health–matched controls. We further aimed to explore the association of several factors with elder-care use and living arrangements among hip fracture patients, including cohabitation with a partner, frailty, and socioeconomic position, using multistate modeling.

## METHODS

### Data sources

This study was based on a linkage of several Swedish administrative population registers (see Web Figure 1, available at https://doi.org/10.1093/aje/kwac149). Data on place of residence and international migration were retrieved from the Total Population Register and death dates from the Cause of Death Register. Hip fractures, frailty, and dementia were identified in the National Patient Register (NPR). Cohabitation was identified in the Dwelling Register and educational levels in the Longitudinal Integrated Database for Health Insurance and Labour Market Studies (LISA) and population censuses.

Information on use of elder-care services was based on the Social Service Register (SSR), an administrative register maintained by the National Board of Health and Welfare (22). In Sweden, municipalities are legally obliged to provide publicly funded home care or nursing homes to older individuals in need. These services are allocated according to need and are provided for small fees that are deemed universally affordable. The SSR contains information on all publicly funded, needs-tested geriatric care in Sweden, even care contracted out to private care providers. Depending on individual needs, home care may include both practical help with daily life—for instance, with shopping, meal preparation, or cleaning (“home help”)—and assistance with personal care—for example, with showering or getting dressed. The type of care is reflected by the number of monthly home care hours provided to each person. Sensitivity analyses based on a sample of municipalities in our study suggested that individuals with fewer hours more often receive home help only, while a larger number of hours indicates the additional provision of personal care (23). Among individuals with fewer than 40 hours of home care per month, 61% received personal care, while among those with 40 or more hours, 98% received personal care.

### Study population

All individuals aged 65 years or more who experienced their first hip fracture during 2014 and 2015 were identified in the NPR. Individuals who had a hip fracture between January 1, 1997, and December 31, 2013, and individuals who emigrated from Sweden after their 60th birthday were excluded. In addition, we excluded individuals residing in municipalities that did not report consistently to the SSR or reported unreasonable data during the study period. Although monthly reporting to the SSR has been mandatory for all Swedish municipalities since 2013, gaps in reporting remained during the beginning of our study period. In total, 197 of 290 municipalities—home to 77% of the Swedish population over the age of 65 years—were included in this study. Both hip fracture patients and matched controls were selected from these municipalities.

This study was approved by the regional ethics committee in Stockholm. The board waived the need for patient consent.

### Variables and definitions

Hip fractures were identified through hospitalization records with *International Classification of Diseases, Tenth Revision*, codes S72.0–S72.2 as primary causes of hospitalization. These are commonly used definitions, and previous studies have demonstrated high levels of validity and completeness for hip fracture diagnoses in administrative inpatient registers, including the Swedish NPR (24–29). Care states were based on data obtained from the SSR, measured on the last day of each month, and categorized into 5 groups: no care, home care with less than 40 hours per month, home care with 40 or more hours per month, residence in a nursing home, and death. Care status at baseline was defined as care registered in the month before the matching date (“baseline”).

To account for individuals’ health aside from care status, we calculated a hospitalization-based frailty index using the algorithm developed by Gilbert et al. (30) and, in addition, coded a separate variable for a diagnosis of dementia in the NPR. The Gilbert frailty index is a weighted score of 109 diagnoses developed to capture information on frailty in routinely collected hospital admission data. Scores less than 5 represent low frailty risk; scores between 5 and 15 represent intermediate frailty risk; and scores greater than 15 represent high frailty risk (30). All diagnoses in the NPR during the 10 years before baseline were taken into account. Likewise, dementia was identified through previous diagnoses in the NPR (*International Classification of Diseases, Tenth Revision*, codes F00–F03 and G30) during the 10 years preceding the baseline date. In the multistate models, which included hip fracture patients only, all of whom were hospitalized, diagnoses made during the hip fracture hospital stay were additionally included in the dementia and frailty variables.

We identified each person’s latest educational level as recorded in either LISA (available from 1990) or population censuses (until 1990), distinguishing between compulsory education (up to 9 years) and higher education. Information on educational level was unavailable for 1.6% of the study population, mostly for individuals in the earliest birth cohort; these individuals were categorized as having the lower educational level. Cohabiting partners at the end of the year before baseline were identified in the Dwelling Register. We further identified partners’ dates of death and categorized individuals whose cohabiting partner died before the baseline date as living alone.

### Matching

For each hip fracture patient, an age- and sex-matched control was randomly selected among individuals who were alive and free of hip fracture up to that age, hereafter referred to as “general population controls.” This comparison allowed us to explore the extent to which individuals who experienced a hip fracture differed from their same-aged peers and to display care trajectories in the general population. Hip fracture occurrence was measured in monthly intervals and age in yearly intervals (with an open category for those aged 100 years or more). For example, a woman born in 1920 who experienced a hip fracture in May 2014 was matched to a woman born in 1920 without a hip fracture before or during May 2014. Controls were drawn with replacement and allowed to experience a hip fracture at any time after baseline.

Older individuals who experience a hip fracture might differ from their same-aged peers in the general population—for instance, through a higher prevalence of other diseases and disabilities. To isolate the influence of the hip fracture itself on care trajectories from differences in underlying health and other characteristics, we employed propensity score matching to generate a control group with characteristics similar to those of hip fracture patients. Propensity scores were based on a logistic regression model including age (years; quadratic variable), sex, education, cohabitation, birth country, dementia, and frailty score (4 categories: 0, 0.1–1.9, 2.0–7.9, or >7.9). The regression model was further stratified by care state at baseline, meaning that matching was performed separately among population groups with one of the 4 care statuses. This method increases flexibility, since covariates in the regression model are allowed to vary—for instance, between individuals without care and those living in nursing homes—and additionally assures a balanced distribution of initial care states, which are probably among the strongest predictors of later care trajectories. Based on the estimated propensity score, each hip fracture patient was matched to the closest hip-fracture–free person alive or, in the case of several potential matches, a randomly selected person among them. The propensity-score–matched control group is hereafter referred to as the health-matched control group.

### Multistate models

**Figure 1:**
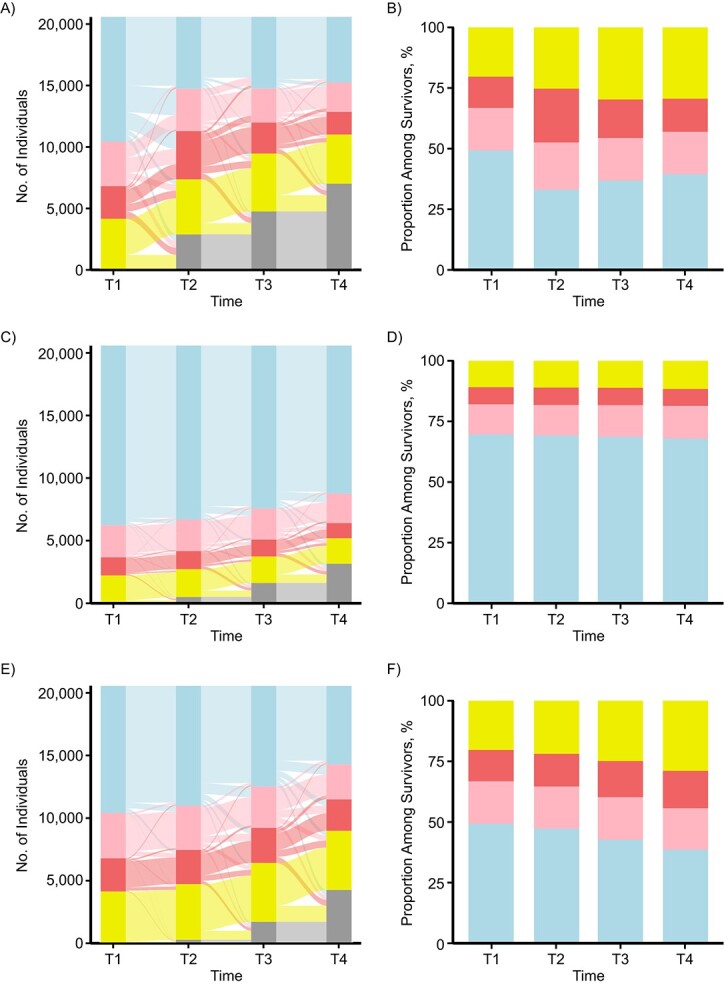
Transitions between care states over the course of 24 months, by time period (T), among hip fracture patients, age- and sex-matched controls in the general population, and propensity-score–matched controls (*n* = 61,719), Sweden, 2014–2017. Left-hand panels show care transitions among hip fracture patients (A), age- and sex-matched controls (C), and health-matched controls (E). Right-hand panels show the care distributions among survivors for hip fracture patients (B), age- and sex-matched controls (D), and health-matched controls (F). T1, baseline; T2, 3 months after baseline; T3, 12 months after baseline; T4, 24 months after baseline. Blue shading: no formal elder care; light red shading: home care for <40 hours/month; dark red shading: home care for ≥40 hours/month; yellow shading: residence in a nursing home; gray shading: death.

**Figure 2:**
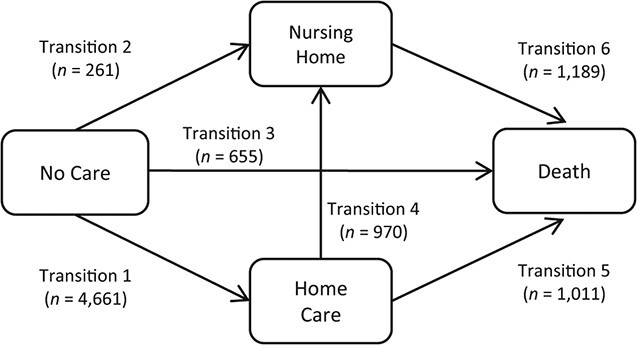
Structure of the multistate model of care-status transitions after experiencing a hip fracture and number of transitions observed within the first 3 months after hip fracture (*n* = 20,573), Sweden, 2014–2017.

Factors associated with different types of care trajectories among hip fracture patients were explored using multistate models (31). This technique allowed us to estimate transition rates between care states while taking into account their interdependence and competing risks. For example, moving into a nursing home, receiving home care, or dying are 3 competing events for community-dwelling individuals without care. To reduce complexity, the 2 home care states were collapsed into one, and the models focused on the time up to 3 months after the fracture. Previous research has showed that most changes in functional abilities among hip fracture patients take place within a few months of the fracture (1), which is also supported by the data shown in Figure 1. Our multistate models allowed for 6 transitions (Figure 2), while individuals who experienced other, rare transitions (e.g., moved from a nursing home to their own home) were assumed to remain in their occupied state until they experienced another transition. Potential predictors included in the analyses were age (5 categories: 65–74, 75–79, 80–84, 85–89, or ≥90 years), sex, cohabitation, educational level, birth country, dementia, and frailty score. Cox regression was used to estimate hazard ratios and 95% confidence intervals for the impact of each covariate on each of the 6 transitions of interest. Estimation was conducted using the packages *mstate* and *survival* (32) in R statistical software, version 3.6.2 (R Foundation for Statistical Computing, Vienna, Austria).

### Sensitivity analyses

We conducted a number of sensitivity analyses to assess the robustness of our findings. Firstly, we explored whether municipalities included in this study differed systematically from those that were not by calculating hip fracture incidence, mean age at hip fracture, and mean frailty score for included and excluded municipalities separately. Secondly, following standard guidelines, we applied a caliper width of 0.2 times the standard deviation of the logit of the propensity score and excluded hip fracture patients who could not be matched to a control (1.2%) from our analyses. Furthermore, we selected an additional control group through propensity score matching without replacement. Finally, instead of including 1 coefficient for each variable and each transition in our multistate model, we used likelihood ratio tests to exclude coefficients for those transitions that did not significantly improve the model fit.

## RESULTS

We identified 20,573 individuals with first hip fracture at a mean age of 83.6 years, 68.1% of whom were women. Table 1 shows the characteristics of these patients, together with those of 20,573 general population controls (age- and sex-matched). Individuals with hip fracture were more likely to live alone, more likely to have a basic education, and more often born in Sweden. Diagnosed dementia was 2.4 times as likely among women with hip fracture and 3.1 times as likely among men with hip fracture than among women and men in the general population. Likewise, a high frailty score was more common among hip fracture patients. At baseline, women more frequently received care than men. However, men had higher mortality during follow-up. Baseline characteristics of hip fracture patients and the health-matched control group were virtually identical (Web Table 1).

**Table 1:** Characteristics (%) of Patients with First Hip Fracture in 2014–2015 and Age-Matched Controls^a^, by Sex (*n* = 41,146), Sweden, 2014–2017

**Characteristic**	**Women**		**Men**	
	**Age-Matched Controls (*n* = 14,010)**	**Hip Fracture Patients (*n* = 14,010)**	**Age-Matched Controls (*n* = 6,563)**	**Hip Fracture Patients (*n* = 6,563)**
Age, years				
≥90^b^	25.0	25.0	18.6	18.6
80–89^c^	46.6	46.6	45.0	45.0
65–79^d^	28.5	28.5	36.4	36.4
Care status at baseline				
No care	66.0	47.4	77.4	53.2
At-home care				
<40 hours/month	13.7	17.8	9.7	16.9
≥40 hours/month	7.9	13.3	5.1	12.1
Residence in a nursing home	12.4	21.5	7.7	17.8
Mortality				
At 1 year	8.1	20.7	7.8	28.7
At 2 years	15.4	31.3	16.5	40.3
Cohabiting	36.7	32.9	66.5	55.7
More than a primary education	47.8	46.8	54.4	51.5
Foreign-born	11.3	9.7	9.9	7.9
Dementia diagnosis^e^	4.7	11.4	4.0	12.3
Frailty status^f^				
Low risk (score ≤4.9)	83.8	72.6	84.5	65.7
Intermediate risk (score 5.0–15.0)	14.3	23.1	13.2	28.5
High risk (score >15.0)	1.9	4.4	2.3	5.8

^a^ Baseline descriptive data are not shown for health-matched controls, since these participants were selected to be similar to hip fracture patients and therefore had almost identical characteristics. Descriptive data for the health-matched control group are shown in Web Table 1.

^b^ Born before 1925.

^c^ Born in 1925–1934.

^d^ Born in 1935 or later.

^e^ Identified in the National Patient Register for the 10 years preceding the hip fracture.

^f^ Frailty was measured by the Gilbert frailty index (30), based on hospitalizations during the 10 years before the hip fracture date or the matching date (excluding diagnoses first made at the time of hip fracture).

### Care trajectories

Figure 1 shows trajectories of long-term care among hip fracture patients and for both control groups. The left-hand panels illustrate longitudinal trajectories from baseline (T1) to 24 months after the fracture (T4), while the right-hand panels present the prevalence of each state at each time point among individuals who survived (i.e., excluding those who died). With regard to mortality, 14.2% of hip fracture patients but less than 3% of both control groups died within 3 months. Cumulative mortality after 2 years among hip fracture patients, general population controls, and health-matched controls was 34.2%, 15.8%, and 20.9%, respectively.

More than half of hip fracture patients but only one-third of the general population control group received any municipal care at baseline, with 20% of hip fracture patients and 11% of controls living in nursing homes. Experiencing a hip fracture had a considerable impact on care statuses, particularly during the first months after the fracture. Almost half of patients without care before their hip fracture received municipal care 3 months after fracture (47.1%) and less than one-quarter of those (23.8%) returned to living without care during follow-up. In contrast, 7.8% of the general population and 10.9% of health-matched controls experienced changes in care status within 3 months after baseline. At T2, two-thirds of hip fracture patients and half of health-matched controls received municipal care despite identical care statuses at baseline. However, 2 years after baseline (T4), the distribution of care states among hip fracture survivors was virtually identical to that of survivors from the health-matched control group.

It is noteworthy that among the general population controls, the shares of individuals living at home, receiving home care, and living in nursing homes were almost identical throughout the study period, whereas we observed a gradual deterioration over time among health-matched controls (Figure 1). Among all groups, transition probabilities were highest for the transition from care-home residence to death.

### Predictors of care status transitions among hip fracture patients

**Figure 3:**
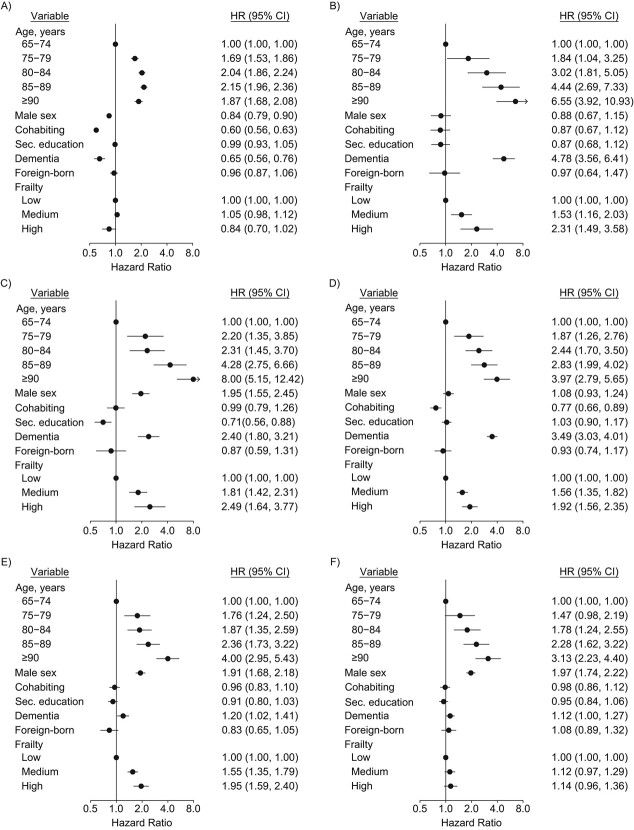
Hazard ratios (HRs) and 95% confidence intervals (CIs; bars) for care-status transitions during the 3 months after hip fracture among 20,573 individuals with hip fracture, Sweden, 2014–2017. Results are shown for transitions from no care to at-home care (A), no care to residence in a nursing home (B), no care to death (C), at-home care to residence in a nursing home (D), at-home care to death (E), and residence in a nursing home to death (F). Frailty was based on the Gilbert frailty index (30). Scores of 4.9 or less indicate low frailty risk; scores of 5.0–15.0 indicate intermediate frailty risk; and scores over 15.0 indicate high frailty risk. Sec., secondary.

Figure 2 shows the number of care-status transitions observed in our multistate framework. In total, there were 8,747 transitions among the 20,573 hip fracture patients. Aside from advanced age, which was consistently associated with higher elder-care use as well as mortality, we identified several risk factors for transitions between care states (Figure 3). Men had consistently higher mortality than women. However, among individuals living without municipal care before their fracture, men were less likely to transition to receiving home care than women. Cohabiting individuals had a lower risk of transitioning from living without care to home care and transitioning from home care to care-home admission. Higher education was associated with a lower risk of death, but only among those living without care. Birth country was not associated with any transition.

Higher frailty scores were associated with increased rates of care-home admission and death both among individuals with home care and individuals without care. By contrast, higher frailty scores were not associated with the transition to home care for individuals without care and were only weakly correlated with higher mortality among care-home residents. Having a dementia diagnosis was, aside from age, the strongest predictor of transitioning into a nursing home and was also associated with higher mortality. However, among individuals without municipal care, dementia was associated with lower rates of transition to home care as compared with individuals without dementia. None of our sensitivity analyses produced results that differed notably from our main findings.

## DISCUSSION

Hip fractures are life-changing conditions that cause a sudden and severe disruption of everyday life. In this study, we showed that individuals who experience a hip fracture are not representative of older men and women in the Swedish population. Instead, they are frailer, more often receive elder-care services, and are already at higher risk of death before sustaining a hip fracture. Yet, even when controlling for prefracture health and care use, our study showed that fracture itself has a tremendous impact on both mortality and care trajectories, particularly during the first months after the fracture. Fourteen percent of individuals with hip fracture died within 3 months as compared with 2% of individuals with characteristics similar to hip fracture patients identified through propensity score matching.

The trajectories shown in this study indicate that increases in care use after hip fracture often persist for several years. Among hip fracture patients without any municipal care at baseline, 40% received home care 3 months later, and the vast majority of them continued to use care after 2 years. Although mortality was high among hip fracture patients, we observed a clear deterioration even among survivors. Almost all hip fracture patients are surgically treated and temporarily immobilized, which can trigger a deterioration of muscle mass and physical strength that may, especially among the oldest old, be difficult to recover from, hence resulting in long-term functional impairment. The fact that individuals who experience a fracture are already frailer and more often care-dependent than their peers before their fracture may also point towards poor resilience and rehabilitation prospects. In addition, hip fracture patients are at high risk of complications, such as infections, hospital readmission, or cardiovascular events (11–13, 33–37). Such complications may increase not only short-term mortality risk but also long-term care needs owing to prolonged immobilization and interrupted rehabilitation.

After 2 years, however, we found that hip fracture patients were at comparable risks of receiving home care, moving to nursing homes, and death as their peers who had similar health and demographic profiles at baseline. This suggests that the impact of a hip fracture on care needs is limited to a relatively short period after the fracture, while long-term care is determined by other factors unrelated to the fracture itself. While hip fracture patients experienced an accelerated decline in health, the health-matched control group seemed to “catch up” with them eventually. Still, one should note that this comparison was conditioned on 2-year survival and considerably more hip fracture patients died within a few months after baseline.

In line with our results, a large body of evidence has documented high excess mortality associated with hip fractures which is most pronounced during the first months after the fracture (5–8) and substantial proportions of patients being admitted to nursing homes (1, 14, 15). However, these studies were often limited to clinical cohorts of community-dwelling individuals and did not consider the use of home care, which is of growing importance for geriatric care in Sweden and many other countries (38, 39). To our knowledge, this is the first populationwide study on living arrangements both before and after hip fracture in comparison with the general population and in comparison with individuals with similar health and living arrangements who did not experience a hip fracture.

To our knowledge, this is also the first study to have examined factors associated with changes in long-term care among hip fracture patients in a multistate model, thereby estimating transitions between several possible care states in a single framework. Van der Sijp et al. (40) recently employed multistate models to identify factors associated with short-term recovery of independence in a smaller clinical cohort of community-dwelling hip fracture patients in the Netherlands. In contrast to our work, those authors did not distinguish between long-term care states and did not report the impact of variables on different transitions.

Previous research identified a variety of predictors for the prognosis of hip fracture patients, including treatment-related factors (2), socioeconomic position (16, 17), social support (16), and comorbidity (2, 18–21, 40). Our analyses indicated that many previously described factors may have heterogenous effects on different care transitions. For instance, it is well-known that men have higher mortality after a hip fracture than women (6, 29), but our work suggests lower rates of transitioning to home care after a hip fracture. This may be partly explained by the higher competing risk of death among men, but in our data, men were less likely to receive home care even after exclusion of those who died. Another potential explanation may be the availability of support by a wife; wives are often younger than their husbands (41) and perhaps take on the traditional caregiver role more naturally. However, the lower risk of receiving home care among men remained even when controlling for cohabitation. We also find dementia to be associated with a lower risk of transitioning to home care, which, again, may be influenced by higher risks of death as well as care-home admission. One should note that in our study, dementia was measured through diagnoses in hospital records. Even though all hip fracture patients were hospitalized and dementia should be registered as a codiagnosis, the sensitivity of this assessment method is probably not perfect. Birth country did not have a significant impact on any care transitions in our study. Most older immigrants living in Sweden today were born in the other Nordic countries and have been exposed to similar genetic, environmental, and socioeconomic risk factors as native Swedes. Considering the increase in international immigration from other areas of the world, individuals experiencing a hip fracture will potentially become a more heterogeneous patient group in the future.

Our study investigated trajectories of long-term geriatric care in Sweden, a setting with high hip fracture incidence (42) and publicly funded care accessible to all residents. Thus, some of our findings may not be generalizable to other countries in which care systems rely more heavily upon family support or nursing homes or settings where home care services come with substantial costs for the individual. We found that mortality of care-home residents was high among both hip fracture patients and controls. This likely reflects the Swedish care system, which aims to allow older individuals to “age in place”; only if needs can no longer be met in an individual’s own home are they transferred to a nursing home. Nursing homes are hence reserved for the frailest and most impaired individuals, who consequently are at higher risk of death.

Strengths of this study include its population-based design, the large study population, and comparison with 2 population-based control groups taking into account prefracture health. This allowed us to explore how hip fracture patients differ from the general population and, moreover, to isolate the impact of the hip fracture on care needs from other factors. Furthermore, we applied multistate models to examine complex care trajectories among hip fracture patients. Our work also had some limitations. Firstly, the hospitalization-based frailty score is a proxy variable and is not a perfect measure of health status and functional abilities. Thus, our matching procedure may not have accounted for all differences between hip fracture patients and the general population. Still, the stratified matching eliminated any differences in the arguably strongest predictor of care trajectories—baseline care status. Secondly, owing to inconsistent reporting to the SSR, we could not include all Swedish municipalities in this study. However, hip fracture patients in excluded municipalities were similar to those in our study, and care is given according to the same principles in all municipalities within Sweden. Lastly, we had no data on informal care or privately purchased services. Although municipal long-term care constitutes more than 90% of formal old age care in Sweden (43–45) and is used by the majority of Swedes at the end of their life (46), informal support provided by close kin is increasing (43, 47). Thus, the care trajectories examined in this study do not necessarily reflect trajectories of functional impairments.

Individuals who experienced a hip fracture were frailer and more likely to receive elder care already before the hip fracture than the general population of the same age. However, even when controlling for prefracture health and care use, hip fractures had a considerable negative impact on mortality, care use, and living arrangements—and patients rarely returned to their prefracture state. Yet, after 2 years, care use was virtually identical among hip fracture survivors and health-matched controls. This suggests that the increase in care use among hip fracture patients would also have occurred in the absence of the fracture, even if somewhat later. Our findings also emphasize the importance of adequate comparison groups when examining the consequences of diseases which are often accompanied by other underlying health problems.

## Supplementary Material

Web_Material_kwac149

## References

[1] Dyer SM , Crotty M, Fairhall N, et al. A critical review of the long-term disability outcomes following hip fracture. *BMC Geriatr*. 2016;16(1):158. 27590604 10.1186/s12877-016-0332-0 PMC5010762

[2] Alexiou KI , Roushias A, Varitimidis SE, et al. Quality of life and psychological consequences in elderly patients after a hip fracture: a review. *Clin Interv Aging*. 2018;13:143–150. 29416322 10.2147/CIA.S150067 PMC5790076

[3] Peeters CMM , Visser E, Van de Ree CLP, et al. Quality of life after hip fracture in the elderly: a systematic literature review. *Injury*. 2016;47(7):1369–1382. 27178770 10.1016/j.injury.2016.04.018

[4] Bertram M , Norman R, Kemp L, et al. Review of the long-term disability associated with hip fractures. *Inj Prev*. 2011;17(6):365–370. 21486987 10.1136/ip.2010.029579

[5] Abrahamsen B , van Staa T, Ariely R, et al. Excess mortality following hip fracture: a systematic epidemiological review. *Osteoporos Int*. 2009;20(10):1633–1650. 19421703 10.1007/s00198-009-0920-3

[6] Haentjens P , Magaziner J, Colón-Emeric CS, et al. Meta-analysis: excess mortality after hip fracture among older women and men. *Ann Intern Med*. 2010;152(6):380–390. 20231569 10.1059/0003-4819-152-6-201003160-00008 PMC3010729

[7] Michaëlsson K , Nordström P, Nordström A, et al. Impact of hip fracture on mortality: a cohort study in hip fracture discordant identical twins. *J Bone Miner Res*. 2014;29(2):424–431. 23821464 10.1002/jbmr.2029

[8] Katsoulis M , Benetou V, Karapetyan T, et al. Excess mortality after hip fracture in elderly persons from Europe and the USA: the CHANCES Project. *J Intern Med*. 2017;281(3):300–310. 28093824 10.1111/joim.12586

[9] Lunde A , Tell GS, Pedersen AB, et al. The role of comorbidity in mortality after hip fracture: a nationwide Norwegian study of 38,126 women with hip fracture matched to a general-population comparison cohort. *Am J Epidemiol*. 2019;188(2):398–407. 30407488 10.1093/aje/kwy251 PMC6357811

[10] Sheehan KJ , Guerrero EM, Tainter D, et al. Prognostic factors of in-hospital complications after hip fracture surgery: a scoping review. *Osteoporos Int*. 2019;30(7):1339–1351. 31037362 10.1007/s00198-019-04976-x

[11] Chiang C-H , Liu C-J, Chen P-J, et al. Hip fracture and risk of acute myocardial infarction: a nationwide study. *J Bone Miner Res*. 2013;28(2):404–411. 22836505 10.1002/jbmr.1714

[12] Pedersen AB , Ehrenstein V, Szépligeti SK, et al. Hip fracture, comorbidity, and the risk of myocardial infarction and stroke: a Danish nationwide cohort study, 1995–2015. *J Bone Miner Res*. 2017;32(12):2339–2346. 28833527 10.1002/jbmr.3242

[13] Tsai CH , Lin CL, Hsu HC, et al. Increased risk of stroke among hip fracture patients: a nationwide cohort study. *Osteoporos Int*. 2015;26(2):645–652. 25300529 10.1007/s00198-014-2919-7

[14] Cancio JM , Vela E, Santaeugènia S, et al. Long-term impact of hip fracture on the use of healthcare resources: a population-based study. *J Am Med Dir Assoc*. 2019;20(4):456–461. 30287263 10.1016/j.jamda.2018.08.005

[15] Pajulammi HM , Pihlajamäki HK, Luukkaala TH, et al. Pre- and perioperative predictors of changes in mobility and living arrangements after hip fracture—a population-based study. *Arch Gerontol Geriatr*. 2015;61(2):182–189. 26043958 10.1016/j.archger.2015.05.007

[16] Auais M , Al-Zoubi F, Matheson A, et al. Understanding the role of social factors in recovery after hip fractures: a structured scoping review. *Health Soc Care Community*. 2019;27(6):1375–1387. 31446636 10.1111/hsc.12830 PMC7039329

[17] Valentin G , Pedersen SE, Christensen R, et al. Socio-economic inequalities in fragility fracture outcomes: a systematic review and meta-analysis of prognostic observational studies. *Osteoporos Int*. 2020;31(1):31–42. 31471664 10.1007/s00198-019-05143-y

[18] Aigner R , Buecking B, Hack J, et al. Pre-fracture hospitalization is associated with worse functional outcome and higher mortality in geriatric hip fracture patients. *Arch Osteoporos*. 2017;12(1):32. 28349470 10.1007/s11657-017-0327-2

[19] Mitchell R , Harvey L, Brodaty H, et al. Hip fracture and the influence of dementia on health outcomes and access to hospital-based rehabilitation for older individuals. *Disabil Rehabil*. 2016;38(23):2286–2295. 26765956 10.3109/09638288.2015.1123306

[20] Mitchell R , Harvey L, Brodaty H, et al. One-year mortality after hip fracture in older individuals: the effects of delirium and dementia. *Arch Gerontol Geriatr*. 2017;72:135–141. 28628893 10.1016/j.archger.2017.06.006

[21] Seitz DP , Gill SS, Gruneir A, et al. Effects of dementia on postoperative outcomes of older adults with hip fractures: a population-based study. *J Am Med Dir Assoc*. 2014;15(5):334–341. 24524851 10.1016/j.jamda.2013.12.011

[22] Socialstyrelsen . Registret över insatser inom kommunal hälso- och sjukvård. https://www.socialstyrelsen.se/statistik-och-data/register/kommunal-halso-och-sjukvard/. Published 2020. Accessed March 26, 2021.

[23] Meyer AC , Sandström G, Modig K. Nationwide data on home care and nursing home residence: presentation of the Swedish Social Service Register, its content and coverage [published online ahead of print December 29, 2021]. *Scand J Public Health*. (10.1177/14034948211061016). PMC9578086 34965796

[24] Hudson M , Avina-Zubieta A, Lacaille D, et al. The validity of administrative data to identify hip fractures is high—a systematic review. *J Clin Epidemiol*. 2013;66(3):278–285. 23347851 10.1016/j.jclinepi.2012.10.004

[25] Huttunen TT , Kannus P, Pihlajamäki H, et al. Pertrochanteric fracture of the femur in the Finnish National Hospital Discharge Register: validity of procedural coding, external cause for injury and diagnosis. *BMC Musculoskelet Disord*. 2014;15(1):98. 24655318 10.1186/1471-2474-15-98 PMC4026595

[26] Lofthus CM , Cappelen I, Osnes EK, et al. Local and national electronic databases in Norway demonstrate a varying degree of validity. *J Clin Epidemiol*. 2005;58(3):280–285. 15718117 10.1016/j.jclinepi.2004.07.003

[27] Ludvigsson JF , Andersson E, Ekbom A, et al. External review and validation of the Swedish national inpatient register. *BMC Public Health*. 2011;11(1):450. 21658213 10.1186/1471-2458-11-450 PMC3142234

[28] Meyer AC , Hedström M, Modig K. The Swedish hip fracture register and National Patient Register were valuable for research on hip fractures: comparison of two registers. *J Clin Epidemiol*. 2020;125:91–99. 32505739 10.1016/j.jclinepi.2020.06.003

[29] Meyer AC , Ek S, Drefahl S, et al. Trends in hip fracture incidence, recurrence, and survival by education and comorbidity: a Swedish register-based study. *Epidemiology*. 2021;32(3):425–433. 33512961 10.1097/EDE.0000000000001321 PMC8011509

[30] Gilbert T , Neuburger J, Kraindler J, et al. Development and validation of a hospital frailty risk score focusing on older people in acute care settings using electronic hospital records: an observational study. *Lancet*. 2018;391(10132):1775–1782. 29706364 10.1016/S0140-6736(18)30668-8 PMC5946808

[31] Putter H , Fiocco M, Geskus RB. Tutorial in biostatistics: competing risks and multi-state models. *Stat Med*. 2007;26(11):2389–2430. 17031868 10.1002/sim.2712

[32] de Wreede LC , Fiocco M, Putter H. The mstate package for estimation and prediction in non- and semi-parametric multi-state and competing risks models. *Comput Methods Programs Biomed*. 2010;99(3):261–274. 20227129 10.1016/j.cmpb.2010.01.001

[33] Meyer AC , Eklund H, Hedström M, et al. The ASA score predicts infections, cardiovascular complications, and hospital readmissions after hip fracture—a nationwide cohort study. *Osteoporos Int*. 2021;32(11):2185–2192. 34013459 10.1007/s00198-021-05956-w PMC8563539

[34] Ali AM , Gibbons CER. Predictors of 30-day hospital readmission after hip fracture: a systematic review. *Injury*. 2017;48(2):243–252. 28063674 10.1016/j.injury.2017.01.005

[35] Ekström W , Samuelsson B, Ponzer S, et al. Sex effects on short-term complications after hip fracture: a prospective cohort study. *Clin Interv Aging*. 2015;10:1259–1266. 26347328 10.2147/CIA.S80100 PMC4531035

[36] Lv H , Yin P, Long A, et al. Clinical characteristics and risk factors of postoperative pneumonia after hip fracture surgery: a prospective cohort study. *Osteoporos Int*. 2016;27(10):3001–3009. 27241669 10.1007/s00198-016-3624-5

[37] Hedström M , Gröndal L, Ahl T. Urinary tract infection in patients with hip fractures. *Injury*. 1999;30(5):341–343. 10505128 10.1016/s0020-1383(99)00094-7

[38] Schön P , Lagergren M, Kåreholt I. Rapid decrease in length of stay in institutional care for older people in Sweden between 2006 and 2012: results from a population-based study. *Health Soc Care Community*. 2016;24(5):631–638. 25944315 10.1111/hsc.12237

[39] Alders P , Schut FT. Trends in ageing and ageing-in-place and the future market for institutional care: scenarios and policy implications. *Health Econ Policy Law*. 2019;14(1):82–100. 29779497 10.1017/S1744133118000129

[40] van der Sijp MPL , van Eijk M, Niggebrugge AHP, et al. Prognostic factors for short-term recovery of independence in a multistate model for patients with a hip fracture. *J Am Med Dir Assoc*. 2021;22(6):1307–1312. 32962930 10.1016/j.jamda.2020.08.006

[41] Drefahl S . How does the age gap between partners affect their survival? *Demography*. 2010;47(2):313–326. 20608099 10.1353/dem.0.0106 PMC3000022

[42] Kanis JA , Odén A, McCloskey EV, et al. A systematic review of hip fracture incidence and probability of fracture worldwide. *Osteoporos Int*. 2012;23(9):2239–2256. 22419370 10.1007/s00198-012-1964-3 PMC3421108

[43] Ulmanen P , Szebehely M. From the state to the family or to the market? Consequences of reduced residential eldercare in Sweden. *Int J Soc Welf*. 2015;24(1):81–92.

[44] Szebehely M , Meagher G. Nordic eldercare—weak universalism becoming weaker? *J Eur Soc Policy*. 2018;28(3):294–308.

[45] Moberg L . Marketisation of Nordic eldercare—is the model still universal? *J Soc Policy*. 2017;46(3):603–621.

[46] Meinow B , Wastesson JW, Kåreholt I, et al. Long-term care use during the last 2 years of life in Sweden: implications for policy to address increased population aging. *J Am Med Dir Assoc*. 2020;21(6):799–805. 32081681 10.1016/j.jamda.2020.01.003

[47] Dahlberg L , Berndt H, Lennartsson C, et al. Receipt of formal and informal help with specific care tasks among older people living in their own home. National trends over two decades. *Soc Policy Admin*. 2018;52(1):91–110.

