# Supplementary material for: The Impact of Hip Fracture on Geriatric Care and Mortality Among Older Swedes: Mapping Care Trajectories and Their Determinants
Source: Am J Epidemiol. 2022 Aug 15;192(1):41–50. doi: 10.1093/aje/kwac149 (PMC9825727; doi:10.1093/aje/kwac149)
Supplement: Web_Material_kwac149 [file web_material_kwac149.pdf]

WEB MATERIAL

The Impact of Hip Fracture on Geriatric Care and Mortality Among Older Swedes: Mapping Care Trajectories and Their Determinants

Anna C. Meyer, Marcus Ebeling, Sven Drefahl, Margareta Hedström, Stina Ek, Glenn Sandström, and Karin Modig

Contents

Web Figure 1: Data Sources (page 2)

Web Table 1: Baseline characteristics of hip fracture patients and controls identified through propensity score matching (page 3)

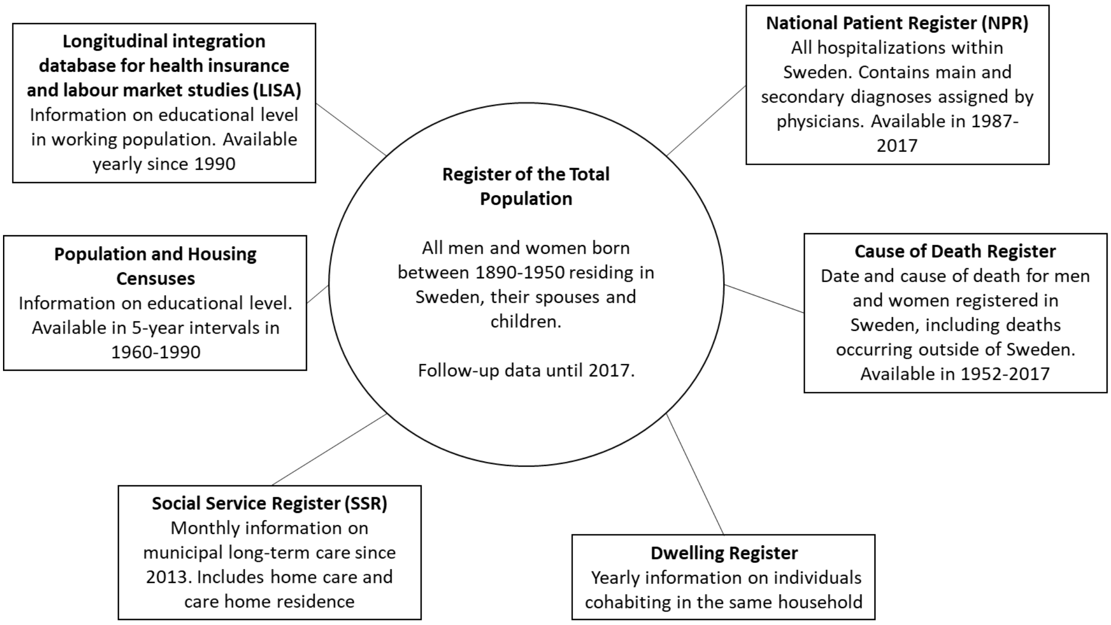

Web Figure 1. Data Sources

Web Table 1. Baseline characteristics of hip fracture patients and controls identified through propensity score matching

	**Matched Controls (*n* = 20,573)**	**Hip Fracture Patients (*n* = 20,573)**
Male (%)	31.5	31.9
Birth year (%)		
<1925	22.9	22.9
1925-1934	46.6	46.1
1935-1950	30.5	31.0
Care status at baseline (%)		
No care	49.2	49.2
Home care <40h	17.5	17.5
Home care 40h+	12.9	12.9
Care home	20.3	20.3
1-year mortality (%)	8.6	25.3
2-year mortality (%)	20.9	36.7
Cohabiting (%)	39.4	40.2
Higher education (%)	48.1	48.3
Born outside of Sweden (%)	7.9	9.1
Prior dementia diagnosis (%)	10.5	11.4
Frailty (%)		
0	18.8	18.8
0.1-1.9	37.7	38.0
2.0-7.9	37.2	36.5
>7.9	6.3	6.8
